# Thematic Analysis of the Health Records of a National Sample of US Veterans With Advanced Kidney Disease Evaluated for Transplant

**DOI:** 10.1001/jamainternmed.2020.6388

**Published:** 2020-11-23

**Authors:** Catherine R. Butler, Aaron Wightman, Claire A. Richards, Ryan S. Laundry, Janelle S. Taylor, Paul L. Hebert, Chuan-Fen Liu, Ann M. O’Hare

**Affiliations:** 1 Division of Nephrology, Department of Medicine, University of Washington, Seattle; 2 Hospital and Specialty Medicine, Geriatrics and Extended Care and Seattle-Denver Health Services Research and Development Center of Innovation, VA Puget Sound Health Care System, Seattle, Washington; 3 Department of Pediatrics, University of Washington School of Medicine, Seattle; 4 Treuman Katz Center for Pediatric Bioethics, Seattle Children’s Hospital, Seattle, Washington; 5 School of Nursing, University of Washington, Seattle; 6 Department of Anthropology, University of Toronto, Toronto, Ontario, Canada; 7 Department of Health Services Research, University of Washington, Seattle

## Abstract

**Importance:**

To be considered for a kidney transplant, patients with advanced kidney disease must participate in a formal evaluation and selection process. Little is known about how this process proceeds in real-world clinical settings.

**Objective:**

To characterize the transplant evaluation process among a representative national sample of US veterans with advanced kidney disease who were referred to a kidney transplant center.

**Design, Setting, and Participants:**

This qualitative study was a thematic analysis of clinician notes in the electronic health records of US veterans referred for kidney transplant evaluation. In a random sample of 4000 patients with advanced kidney disease between January 1, 2004, and December 31, 2014, cared for in the US Department of Veterans Affairs (VA) health care system, there were 211 patients who were referred to a transplant center during the follow-up period. This group was included in the qualitative analysis and was followed up until their date of death or the end of the follow-up period on October 8, 2019.

**Main Outcomes and Measures:**

Dominant themes pertaining to the kidney transplant evaluation and selection process identified through thematic analysis.

**Results:**

Among 211 study patients, the mean (SD) age was 57.9 (9.5) years, and 202 patients (95.7%) were male. The following 4 dominant themes regarding the transplant evaluation process emerged: (1) far-reaching and inflexible medical evaluation, in which patients were expected to complete an extensive evaluation that could have substantial physical and emotional consequences, made little accommodation for their personal values and needs, and impacted other aspects of their care; (2) psychosocial valuation, in which the psychosocial component of the transplant assessment could be subjective and intrusive and could place substantial demands on patients’ family members; (3) surveillance over compliance, in which the patients’ ability and willingness to follow medical recommendations was an important criterion for transplant candidacy and their adherence to a wide range of recommendations and treatments was closely monitored; and (4) disempowerment and lack of transparency, in which patients and their local clinicians were often unsure about what to expect during the evaluation process or about the rationale for selection decisions. For the evaluation process to proceed, local clinicians had to follow transplant center requirements even when they believed the requirements did not align with best practices or the patients’ needs.

**Conclusions and Relevance:**

In this qualitative study of US veterans with advanced kidney disease evaluated for transplant, clinician documentation in the medical record indicated that, to be considered for a kidney transplant, patients were required to participate in a rigid, demanding, and opaque evaluation and selection process over which they and their local clinicians had little control. These findings highlight the need for a more evidence-based, individualized, and collaborative approach to kidney transplant evaluation.

## Key Points

**Question:**

What types of clinical care are involved in the kidney transplant evaluation process in real-world clinical settings?

**Findings:**

In this qualitative study of the electronic health records of 211 US veterans with advanced kidney disease who were referred for kidney transplant evaluation, 4 dominant themes were identified describing clinical care during the evaluation process: far-reaching and inflexible medical evaluation, psychosocial valuation, surveillance over compliance, and disempowerment and lack of transparency.

**Meaning:**

In this study, clinician documentation in the medical record indicated that, to be considered for a kidney transplant, patients were required to participate in a rigid, demanding, and opaque evaluation process over which they and their local clinicians had little control.

## Teaser

This qualitative study describes the real-world experience for patients with advanced kidney disease referred for the kidney transplant evaluation and selection process.

## Introduction

Kidney transplant is a preferred treatment for end-stage kidney disease.^1,2^ However, because there are risks associated with transplant and the supply of donor organs is limited, patients are required to participate in a formal medical and psychosocial evaluation before they can be added to the deceased donor transplant waiting list or can be accepted for a transplant from a living donor. Once selected, patients may spend years on the national waiting list before receiving a transplant from a deceased donor. During this time, they are closely monitored by the transplant center for signs of deteriorating health and/or the development of new contraindications to transplant.

The National Kidney Allocation System relies on an explicit and publicly available algorithm that was developed with input from diverse stakeholders to determine how patients on the waiting list are selected for transplant from a deceased donor.^3^ However, the processes by which transplant center teams evaluate and select patients for inclusion on the deceased donor waiting list and for transplant from a living donor are less transparent.^4,5,6^ Previous studies describing the transplant process have focused on the factors and outcomes associated with referral, waiting list inclusion, and receipt of a kidney transplant^7,8,9,10,11^ and on patients’ experiences with particular aspects of the transplant process.^12,13,14,15,16^ To our knowledge, few previous studies have described how the entire process of referral, evaluation, and selection for kidney transplant proceeds in real-world clinical settings.^17^ A more complete understanding of this process could help to improve the quality of care for patients and families, guide process improvement, and support public discussion about strategies to promote fair allocation of donor kidneys.^5,18^

The US Department of Veterans Affairs (VA) health care system is the only national health care system that supports all steps in the kidney transplant process internally, including referral and evaluation, selection, and surgery. On an annual basis, approximately 1800 patients are referred to 1 of 7 VA kidney transplant centers for evaluation, and approximately 160 kidney transplants are performed within the VA system.^19^ An estimated two-thirds of referred patients are invited to participate in an in-person evaluation, and the remainder are declined as transplant candidates without further evaluation.^20^ Approximately one-half of patients invited for an in-person evaluation at a VA transplant center are selected for inclusion on the national kidney transplant waiting list.^20^

To understand how evaluation and selection for kidney transplant unfolds in real-world clinical settings, we conducted a qualitative study of clinician documentation in the electronic health records (EHRs) of patients with advanced kidney disease who were referred to VA transplant centers.

## Methods

### Cohort Selection and Data Collection

Using a previously published approach,^21,22,23,24^ we conducted a qualitative study of clinician documentation in the EHRs of members of a representative national sample of US veterans with advanced kidney disease who were referred for evaluation at 1 or more VA kidney transplant centers. The study was approved by the VA Central Institutional Review Board with a waiver of informed consent for all study procedures because of minimal risk to patients.

We identified a national cohort of 130 374 patients with advanced kidney disease (defined as an estimated glomerular filtration rate <20 mL/min/1.73 m^2^ on at least 2 occasions at least 3 months apart) between October 1, 1999, and December 31, 2014 (eFigure 1 in the Supplement). We used linked clinical and administrative data from the VA, the Centers for Medicare & Medicaid Services, and the US Renal Data System (a national registry for patients with end-stage kidney disease) to assemble and characterize the study cohort. Patients entered the cohort on the date of their second finding of an estimated glomerular filtration rate of less than 20 mL/min/1.73 m^2^. Patients were followed up until their date of death or the end of the follow-up period on October 8, 2019. We then selected a random sample of 4000 patients who entered the cohort between January 1, 2004, and December 31, 2014, for in-depth medical record review. We obtained all clinician progress notes that were stored in the VA Corporate Data Warehouse between the time of cohort entry and the end of the follow-up period. We used the Veterans Indexed Search for Analysis tool, a Lucene-based text search instrument,^22^ to identify all mentions of the term *transplant* (which included mentions that contained the root word, such as *transplantation* and *transplants*) in note titles or free text in patients’ medical records between the time of cohort entry and the end of the follow-up period.

A text search of the EHRs of 4000 patients yielded 97 515 notes with at least 1 mention of the term *transplant* belonging to 1896 unique patients. One of us (C.R.B., a nephrologist and research fellow) reviewed notes with at least 1 *transplant* mention to exclude the following groups: (1) 481 patients for whom mentions containing the term *transplant* did not pertain to the kidney transplant evaluation process (eg, patients who had received a kidney transplant before cohort entry or patients for whom the *transplant* mention was in reference to a different type of organ transplant or appeared only as part of a standard template element in the medical record) and (2) 1204 patients with a valid *transplant* mention who were not referred to a VA transplant center during the follow-up period. Using this approach, we identified an analytic cohort of 211 patients (5.3%) who were referred to a VA transplant center during the follow-up period.

### Qualitative Analyses

One of us (C.R.B.) reviewed all notes with *transplant* mentions that belonged to the 211 cohort members referred to a VA transplant center and abstracted passages pertaining to the transplant evaluation process from their EHRs. Two of us who are experienced in qualitative methodology (C.R.B. and A.M.O., a physician scientist) used inductive thematic analysis, an approach for analyzing unstructured text that facilitates discovery of previously unidentified factors associated with a phenomenon of interest,^25^ to independently review and openly code abstracted passages for concepts associated with the transplant evaluation. One of us (A.M.O.) reviewed all records for a random selection of 55 cohort members who were referred to a VA transplant center, and another one of us (C.R.B.) reviewed records for all 211 patients. We used an iterative and consensus-based approach to define codes and resolve differences in interpretation until we reached thematic saturation, the point at which no new concepts were identified with additional review.^26,27^ We then collapsed associated codes into larger thematic categories, returning frequently to the primary passages to ensure that emergent themes were grounded in the data.^25,27^ We used ATLAS.ti, version 8 (Scientific Software Development), to organize and store text and to support coding.

### Statistical Analysis

SAS, version 9.4 (SAS Institute) was used to assemble the cohort. Stata, version 15 (StataCorp LLC) was used to describe the characteristics of patients referred for transplant evaluation.

## Results

Among a national sample of 4000 patients with advanced kidney disease who received care in the VA health care system from January 1, 2004, to December 31, 2014, 211 patients were referred to a VA transplant center during follow-up through October 8, 2019. Of those, 202 patients (95.7%) were male, 118 patients (55.9%) were White, and 75 patients (35.5%) were Black or African American; the mean (SD) age of cohort members was 57.9 (9.5) years (Table 1). A total of 51 patients (24.2%) had initiated maintenance dialysis by the time of cohort entry. Nine patients (4.3%) were enrolled in Medicaid in the year before cohort entry. Based on qualitative analysis of notes containing *transplant* mentions, we identified 4 themes related to the transplant process: (1) far-reaching and inflexible medical evaluation, (2) psychosocial valuation, (3) surveillance over compliance, and (4) disempowerment and lack of transparency.

**Table 1.** Characteristics of Patients With Advanced Kidney Disease Who Were Referred to a US Department of Veterans Affairs Kidney Transplant Center

Characteristic	Patients, No. (%) (N = 211)
Age, mean (SD), y	57.9 (9.5)
Race^a^	
White	118 (55.9)
Black or African American	75 (35.5)
Other	13 (6.2)
Unknown	5 (2.4)
Sex	
Male	202 (95.7)
Female	9 (4.3)
Comorbidities in the year before cohort entry	
Diabetes	133 (63.0)
Coronary artery disease	54 (25.6)
End-stage kidney disease	51 (24.2)
Congestive heart failure	43 (20.4)
Cerebrovascular disease	28 (13.3)
Peripheral arterial disease	26 (12.3)
Cancer	19 (9.0)
Chronic obstructive pulmonary disease	0
Enrolled in both Medicare and Medicaid within the year before cohort entry^b^	9 (4.3)
Referral transplant center^c^	
Portland, Oregon	60 (28.4)
Pittsburgh, Pennsylvania	58 (27.5)
Nashville, Tennessee	44 (20.9)
Iowa City, Iowa	39 (18.5)
Houston, Texas	18 (8.5)
Bronx, New York	7 (3.3)
Birmingham, Alabama	3 (1.4)
Unknown^d^	9 (4.3)
Other^e^	2 (0.9)

^a^ Race as documented in Medicare and Department of Veterans Affairs files may not have been based on self-reporting.

^b^ Medicaid enrollment at any time in the year before cohort entry was ascertained from Medicare enrollment files.

^c^ Referral transplant centers were nonexclusive; 23 patients were referred to multiple centers.

^d^ The notes in the electronic health record did not specify the transplant center to which the patient was referred.

^e^ The patients visited a different Department of Veterans Affairs transplant center for consideration of dual-organ transplant.

### Far-reaching and Inflexible Medical Evaluation

Documentation in patients’ EHRs suggested that clinicians approached the transplant evaluation with the goal of fully characterizing patients’ known health conditions and identifying undiagnosed conditions (example quotations are included in Table 2). As part of the evaluation, patients were expected to attend multiple clinic visits with different specialists and might be referred for invasive procedures, such as cardiac catheterization, liver biopsy, dental extractions, and surgeries, many of which would not have been indicated as part of routine care. Testing conducted as part of the transplant evaluation could involve risks and sometimes led to a cascade of further testing and treatments.

**Table 2.** Example Quotations Illustrating the Theme of Far-reaching and Inflexible Medical Evaluation

Source	Example quotation^a^
Urology consultation note	A suspicious renal mass of [this] size normally could be managed with surveillance. However, given that he is on a transplant list and will not be able to get a kidney transplant if he has renal cell carcinoma, definitive management may be necessary.
Discharge summary	In pursuit of renal transplant, he underwent percutaneous liver biopsy on [date] complicated by hemobilia and occult GI bleeding.
Pulmonology clinic note	He had an echo [last year] for transplant workup and moderately elevated RVSP (45-50) was noted...Cardiology wanted pulmonary evaluation. Therefore, a V/Q scan, polysomnogram, and CXR were recommended.
Mental health note	“I am like an old car...they keep on finding more things wrong with me and fixing them.” [in reference to the kidney transplant evaluation]
Nephrology clinic note	He views [peritoneal dialysis] as a means to a potential transplant...We discussed that he would be interested in dialysis as a bridge but was unsure if this would be something he would do otherwise.
Primary care clinic note	TSH mildly elevated, awaiting transplant decision before making dose changes.
Gastroenterology consultation note	He states that a gastric lesion was seen on MRI and he was to have EGD but declined…he is afraid that his workup for renal transplant will be delayed if anything is found.
Social work transplant evaluation note	We discussed his pulmonary nodule…They recommended CT-guided biopsy, but he initially refused this, as he personally feels it’s unlikely that he has cancer, and is willing to assume the risk…[He] wants a kidney transplant, and this issue needs to be resolved before that.
Psychiatric clinic note	The patient presented with a lot of anxiety related to upcoming medical procedures that are prerequisite for a possible kidney transplant...fears regarding “not waking up” from some of these procedures.
Suicide risk prevention assessment	He reported disappointment and exacerbation in depression as a result of being dropped from a list of possible recipients for a kidney transplant. He admitted to suicidal ideation.

Abbreviations: CT, computed tomography; CXR, chest radiograph; EGD, esophagogastroduodenoscopy; GI, gastrointestinal; MRI, magnetic resonance imaging; RVSP, right ventricular systolic pressure; TSH, thyroid-stimulating hormone; V/Q, ventilation-perfusion.

^a^ Nonstandardized abbreviations have been expanded, and misspellings have been corrected.

Participation in the transplant evaluation could also have implications for decisions about other aspects of patients’ care, even when these aspects were not associated with the transplant process. Patients and their clinicians might have delayed or avoided receiving tests or treatments because of concern that these might adversely impact the patients’ transplant candidacy. In general, the transplant evaluation process made little accommodation for the distinct circumstances, priorities, and values of individual patients.

The need for frequent testing and interaction with the health care system during the transplant evaluation process could be overwhelming and emotionally burdensome for some patients and families. Setbacks or deviations from expectations in the evaluation process were sometimes associated with depression and even suicidal ideation.

### Psychosocial Evaluation

Interviews conducted by social workers and mental health specialists as part of the psychosocial assessment included detailed questions about patients’ living arrangements, employment history, substance use, financial status, and legal history as well as in-depth questions about family members (example quotations are included in Table 3). Ongoing substance use (eg, alcohol, tobacco, and marijuana) constituted an unconditional contraindication to transplant regardless of the circumstances or frequency of the substance use. Clinicians’ questions and recommendations sometimes strayed into patients’ personal affairs and could be upsetting or embarrassing. Documentation in medical records suggested that patients’ awareness of clinicians’ conflicting obligations to advocate for them while also being required to report potentially compromising information to the transplant center could stifle honest communication and undermine trust. Referral letters to the transplant center sometimes conveyed a moral tone alluding to whether patients were deserving of transplant.

**Table 3.** Example Quotations Illustrating the Theme of Psychosocial Evaluation

Source	Example quotation^a^
Mental health transplant evaluation note	This patient currently drinks alcohol on a social basis. His last drink of alcohol was 1 mo ago when he drank 1 glass of wine...This patient expressed a willingness to abstain from alcohol use as required and to submit to random screening.
Mental health transplant evaluation note	The patient should consider nourishing his relationship with his wife...Importance of a healthy relationship with his wife prior to and during the transplant process.
Mental health note	Guardedness regarding his substance use history...Upon later discussion he said to her in front of his friend that he didn't like to talk about his past drug use.
Mental health transplant evaluation note	They state that patient’s name has been removed from the kidney transplant list due to information that was in writer's notes…Patient's wife states they never would have discussed any confidential information with writer if they had known that such information would be written in the patient’s medical record.
Physician transplant referral note	[The patient] would make an excellent deceased donor kidney transplant candidate…Because of his youth, combat service (2 tours in Iraq) and excellent functional status this writer feels the patient should be given the highest priority for opportunity for kidney transplantation.
Nephrology clinic note	He is an extremely compliant patient, functional and active, very dedicated to his children...He has been pleading with us to refer him for consideration for transplantation as he has 2 young children with no one else to provide for them.
Psychiatry transplant evaluation note	Long-standing supportive marriage and stable financial circumstances are also good predictors of positive outcome.
Nephrology clinic note	This gentleman appears to be in many ways a “loner”...he states he did not marry, and he is averse to marriage now. This does not of course disqualify him from transplant consideration, but I want to be more sure he does have ex-fiancée’s support and some insight into this dynamic.
Mental health transplant evaluation note	[His wife] reported that her number 1 priority is to support her husband as he goes through the process of transplantation...She indicated that she would quit her job if she was not granted the leave.
Social work transplant evaluation note	He is looking for someone to accompany him and stay with him for at least a month when/if he is approved for a transplant. His wife is disabled so not able to accompany and help him. His mother is in poor health. He has no siblings or children...[the patient] is aware it is unlikely he'll be able to find someone who can put their life on hold for a month without any compensation.

^a^ Nonstandardized abbreviations have been expanded, and misspellings have been corrected.

As part of the transplant process, patients were expected to identify a support person who could care for them during the immediate posttransplant period. Documentation by some transplant specialists suggested a preference for support persons with formal relationships (ie, a spouse or family member) and adequate financial resources. We found examples of support persons who were prepared to relocate and/or quit their jobs to fulfill this responsibility. We also found examples of patients who could not be accepted as transplant candidates because they did not have family or friends who were willing and able to fulfill this role.

### Surveillance Over Compliance

In addition to completing the medical and psychosocial evaluation, patients were expected to demonstrate personal responsibility for improving their health and social circumstances (example quotations are included in Table 4). The extent to which a patient adhered to treatment recommendations could be viewed by clinicians as an indication of the patient’s level of motivation to receive a kidney transplant and as a “litmus test” for how the patient would fare after transplant. We also found examples of clinicians encouraging patients to adhere to treatment recommendations by reminding them that adherence could impact their transplant candidacy. Clinicians at both the local medical center and the transplant center routinely reviewed patients’ medical records to assess the extent to which they were adhering to clinical recommendations and to identify risk factors for nonadherence. Missed clinic appointments and missed or shortened dialysis treatments were interpreted as indicators of “noncompliance” with little consideration for extenuating circumstances or the values and preferences of individual patients. Nonadherence to prescribed medications raised questions about whether patients would be able and willing to adhere to posttransplant medication regimens. However, clinicians tracked and encouraged adherence to a range of other medical recommendations that appeared to be less relevant to posttransplant care.

**Table 4.** Example Quotations Illustrating the Theme of Surveillance Over Compliance

Source	Example quotation^a^
Physician letter to patient	I told him that he was the “driver” of the completion of the tests to get on the transplant list and he needs to keep me informed of the results.
Psychiatry clinic note	[The patient] is feeling a little ambivalent about having a transplant evidenced by his lack of interest in agreeing to a 10-pound weight loss.
Physician note	The entire transplant evaluation is somewhat of a litmus test for [the patient] to see if he can follow through appropriately with each step.
Transplant center letter to patient	We have changed your listing status on the UNOS kidney transplant wait list to an inactive status as of [date]. We’re very concerned about your present compliance and your willingness/ability to comply with the transplant regimen following transplantation.
Dialysis nurse note	Patient was told that if he continues noncompliance with medications, he will likely end up back on hemodialysis and also is jeopardizing his chances for transplantation.
Transplant coordinator note	[The patient] has a 28% compliance/response rate to the Home Telehealth Program…[transplant] Care Coordinator informed him that there is a national requirement to participate 70% of the time, or more often.
Nephrology clinic note	At this point I would be concerned that he misses too much dialysis and is well over a year in getting [hemodialysis vascular] access done to be good transplant candidate (compliance red flags).
Mental health transplant evaluation note	He was called noncompliant by health providers but disagrees with the [hemodialysis vascular] access points, location in the arm, because of collapsed veins and not wanting to be disfigured with nodules in his arms.
Transplant surgery note	This noncompliance with medications is particularly concerning in regard to transplantation since consistent dosing of medications is critical to prevent rejection of the transplanted organ.
Transplant center staff note	Of note, when he arrived at this VA, after checking in, he went to a nearby casino without alerting the floor. He arrived back at the hospital quite late.…He was cautioned about such behavior being perceived as noncompliant.

Abbreviations: UNOS, United Network for Organ Sharing; VA, US Department of Veterans Affairs.

^a^ Nonstandardized abbreviations have been expanded, and misspellings have been corrected.

### Disempowerment and Lack of Transparency

Patients often began the transplant evaluation around the same time that they started or were preparing to start dialysis treatment. When facing the prospect or experiencing the reality of long-term dialysis treatment, some patients expressed a willingness to do anything to avoid having to rely on dialysis for the remainder of their lives (example quotations are included in Table 5).

**Table 5.** Example Quotations Illustrating the Theme of Disempowerment and Lack of Transparency

Source	Example quotation^a^
Social work transplant evaluation note	Appears to be compliant. States that he would “do anything to keep from keeping on dialysis.”
Cardiology consultation note	There is good evidence that preoperative revascularization even in patients with high risk stress results does not significantly decrease the risk of peri-operative ischemic events (eg, DECREASE V trial)...we have become reluctant to perform cardiac catheterization solely for this reason. We are well aware that the criteria for listing the patient for a renal transplant are different.
Pharmacy note	Per [VA transplant center], patient's A1_c_ is to be less than 7% prior to transplant…Would wonder if obtaining goal of less than 7% may be harmful to patient due to already present hypoglycemic episodes.
Social work telephone note	He has no transplant evaluation appointments scheduled as of this writing. Patient was under the impression that he is already listed.
Hepatology clinic note	Liver biopsy would be indicated to sort this out as it might change plans in terms of renal transplant. The couple wanted to know more about this and I asked them to talk to their nephrologist, [name], or [transplant coordinator] about [if] in the event that we find cirrhosis that would disqualify him for a renal transplant, as I was not clear of the answer of this.
Social work telephone note	He questioned this decision [declined transplant candidacy], stating, “I don't understand” and explains that he has been steadfast in making it to all of his appointments...”I've jumped through hoops and done everything they've asked.”
Transplant center letter to patient	The kidney transplant team at the [VA transplant center] has determined that kidney transplantation would not be in your best interest and you will be removed from the transplant [waiting] list.
Transplant coordinator note	Transplant center #2 has turned him down, [the patient] wants to know is there anywhere else to appeal. He states he would rather have a chance of cancer reoccurrence than stay on dialysis.
Mental health transplant evaluation note	He described his hopeless outlook about getting a kidney transplant: “Somewhere in Washington [District of Columbia] some person behind a desk is going to be looking at my file and say, do we even want to give it to this guy? It is like people just get fed up with me and don't want to help me. It is just like how you (ie, this writer) get when I don't do what you want.”
Dialysis physician note	He became quite anxious on [date], shouting, “they just gonna let me die; they not going to give me a kidney because I smoke?!”

Abbreviations: A1_c_, glycated hemoglobin; VA, US Department of Veterans Affairs.

^a^ Nonstandardized abbreviations have been expanded, and misspellings have been corrected. Text in brackets was added by us to clarify and/or replace identifying information in the original text.

To help patients move forward with the transplant process, clinicians at the patient’s local medical center sometimes had to assume a more technical role, performing tests and administering treatments required by the transplant center even when these did not make sense to them, deviated from perceived best practices, or could be harmful.

Patients, family members, and local clinicians were often unsure or mistaken about the patient’s status in the transplant process. Documentation from specialists responsible for discrete aspects of the evaluation suggested that they did not always know how the results of their testing might impact a patient’s transplant candidacy. Clinician documentation suggested that patients were sometimes surprised to find that their candidacy had been declined by the transplant center.

Documentation from the transplant center rarely made explicit mention of the team’s role as stewards of a scarce resource. Letters to patients and referring clinicians focused instead on the transplant team’s assessment of the risk of negative outcomes and described what the team had determined to be in the best interests of individual patients. Transplant center assessments took little account of the priorities of individual patients and their willingness to assume risk. Clinicians’ notes in the medical record sometimes described patients’ distress at what they perceived to be arbitrary or unfair decisions about their transplant candidacy.

## Discussion

Our thematic analysis of the medical records of a national sample of patients with advanced kidney disease who were referred for kidney transplant evaluation provides insights into how this process proceeds in real-world clinical settings. Because kidney transplant was a desirable goal for patients, they had little choice but to participate in an evaluation and selection process that could be inflexible and demanding and over which they and their local clinicians had little control.

The need to formally incorporate societal considerations that go beyond the needs of individual patients (eg, fair allocation of a scarce resource) distinguishes the evaluation and selection process for kidney and other types of solid organ transplants from most other clinical care processes. Nonetheless, there is general agreement that this process should strive to uphold patients’ goals, values, and preferences to the greatest extent possible.^28^ Our analysis suggests that there are opportunities to make the evaluation process more person-centered. Documentation in the medical record suggested that patients and their local clinicians were often unsure about what to expect from the evaluation process or about the rationale for evaluation requirements or selection decisions, which may impede shared decision-making.^12,29^ There was also little accommodation for the needs of patients and families and few opportunities for patients’ local clinicians to use their clinical judgment and provide individualized care.

Transplant centers are given substantial flexibility to define their own systems for evaluating and selecting transplant candidates with the aim of being responsive to the particular needs of the populations they serve.^28^ However, this approach can produce variability in the selection and evaluation processes across centers^30,31,32,33^ and limit transparency, accountability, and opportunities for stakeholder input, features that are critical for ensuring a fair allocation system.^4,5,6^ The use of subjective and non–evidence-based selection criteria^34,35,36^ and the rigidity with which these criteria are applied may have the unintended consequence of leaving patients and families vulnerable to the harms of overtesting,^37^ implicit bias,^38,39^ and discriminatory practices.^40,41^ In particular, we suspect that requirements for complete abstinence from substances (including legal substances),^42^ narrow definitions of acceptable social support,^43,44^ and insistence on “compliance” with a wide range of medical recommendations^45^ may have disproportionately impacted the most disadvantaged members of the cohort.

Our findings highlight the need for a more person-centered and equitable approach to the kidney transplant evaluation and selection process (eFigure 2 in the Supplement). An evaluation and selection process that is more substantially guided by high-quality evidence and communally accepted principles of fairness and social justice could limit the burden of irrelevant testing procedures and protect patients from harmful, arbitrary, and/or discriminatory evaluation and selection practices.^37,40,46^ More open communication and collaboration between the transplant center and patients’ local medical centers could also allow for greater transparency and flexibility in the evaluation process. A collaborative approach could empower local clinicians to individualize the evaluation process within established parameters to accommodate the needs and circumstances of individual patients and families.^47^ Greater transparency in the transplant evaluation and selection process could also serve to improve public accountability and trust^5,18^ and support shared decision-making by providing patients and their families with more realistic expectations about the evaluation.^47^

### Strengths and Limitations

This study has several strengths and limitations. The national scope and integrated nature of the VA health care system allowed us to identify a representative sample of patients who were referred for transplant evaluation and to track their involvement from an early stage in the transplant process. However, because of the distinctive organizational structure of the VA transplant program, some of the themes that emerged from this analysis may not be transferable to other health systems or to groups of patients who are not well represented in the VA population, such as women and young adults. Some of our findings (eg, uncertainty about transplant status) are particularly notable because the VA system has an organizational policy regarding kidney transplant evaluation that is more explicit, centralized, and accountable than that of many other health care organizations.^19,48^ This study is also limited because not all aspects of clinical encounters were documented in the medical record and medical record documentation does not provide direct information about the experiences or perspectives of patients, families, or clinicians.

## Conclusions

This qualitative analysis of documentation in the EHRs of a representative national sample of US veterans with advanced kidney disease who were referred to a VA transplant center suggests that there are opportunities to make the transplant evaluation process more person-centered and equitable. Our findings highlight the need for a more evidence-based, individualized, and collaborative approach to evaluation and selection for kidney transplant.

## Notes

Supplement. **eFigure 1.** Cohort Derivation **eFigure 2.** Diagram of Emergent Themes

## References

[1] Wolfe RA, Ashby VB, Milford EL, Comparison of mortality in all patients on dialysis, patients on dialysis awaiting transplantation, and recipients of a first cadaveric transplant. N Engl J Med. 1999;341(23):1725-1730. doi:10.1056/NEJM199912023412303 10580071

[2] Legeai C, Andrianasolo RM, Moranne O, Benefits of kidney transplantation for a national cohort of patients aged 70 years and older starting renal replacement therapy. Am J Transplant. 2018;18(11):2695-2707. doi:10.1111/ajt.15110 30203618

[3] Friedewald JJ, Samana CJ, Kasiske BL, The kidney allocation system. Surg Clin North Am. 2013;93(6):1395-1406. doi:10.1016/j.suc.2013.08.007 24206858

[4] Volk ML, Biggins SW, Huang MA, Argo CK, Fontana RJ, Anspach RR Decision making in liver transplant selection committees: a multicenter study. Ann Intern Med. 2011;155(8):503-508. doi:10.7326/0003-4819-155-8-201110180-00006 22007044 PMC3197782

[5] Boulware LE, Troll MU, Wang N-Y, Powe NR Perceived transparency and fairness of the organ allocation system and willingness to donate organs: a national study. Am J Transplant. 2007;7(7):1778-1787. doi:10.1111/j.1600-6143.2007.01848.x 17524080

[6] Caplan AL Organ transplant rationing: a window to the future? Health Prog. 1987;68(5):40-45. 10282293

[7] Alexander GC, Sehgal AR Barriers to cadaveric renal transplantation among blacks, women, and the poor. JAMA. 1998;280(13):1148-1152. doi:10.1001/jama.280.13.1148 9777814

[8] Patzer RE, Plantinga LC, Paul S, Variation in dialysis facility referral for kidney transplantation among patients with end-stage renal disease in Georgia. JAMA. 2015;314(6):582-594. doi:10.1001/jama.2015.8897 26262796 PMC4571496

[9] Bloembergen WE, Mauger EA, Wolfe RA, Port FK Association of gender and access to cadaveric renal transplantation. Am J Kidney Dis. 1997;30(6):733-738. doi:10.1016/S0272-6386(97)90076-7 9398115

[10] Kim SJ, Gill JS, Knoll G, Referral for kidney transplantation in Canadian provinces. J Am Soc Nephrol. 2019;30(9):1708-1721. doi:10.1681/ASN.2019020127 31387925 PMC6727260

[11] Hart A, Smith JM, Skeans MA, OPTN/SRTR 2018 annual data report: kidney. Am J Transplant. 2020;20(suppl s1):20-130. doi:10.1111/ajt.15672 31898417

[12] Tong A, Hanson CS, Chapman JR, ‘Suspended in a paradox’—patient attitudes to wait-listing for kidney transplantation: systematic review and thematic synthesis of qualitative studies. Transpl Int. 2015;28(7):771-787. doi:10.1111/tri.12575 25847569

[13] Spiers J, Smith JA Waiting for a kidney from a deceased donor: an interpretative phenomenological analysis. Psychol Health Med. 2016;21(7):836-844. doi:10.1080/13548506.2015.1112415 26584590

[14] Martin SC, Stone AM, Scott AM, Brashers DE Medical, personal, and social forms of uncertainty across the transplantation trajectory. Qual Health Res. 2010;20(2):182-196. doi:10.1177/1049732309356284 19955227

[15] Burns T, Fernandez R, Stephens M The experiences of adults who are on dialysis and waiting for a renal transplant from a deceased donor: a systematic review. JBI Database System Rev Implement Rep. 2015;13(2):169-211. doi:10.11124/jbisrir-2015-1973 26447040

[16] Pinter J, Hanson CS, Chapman JR, Perspectives of older kidney transplant recipients on kidney transplantation. Clin J Am Soc Nephrol. 2017;12(3):443-453. doi:10.2215/CJN.05890616 28143863 PMC5338704

[17] Butler CR, Taylor JS, Reese PP, O’Hare AM Thematic analysis of the medical records of patients evaluated for kidney transplant who did not receive a kidney. BMC Nephrol. 2020;21(1):300. doi:10.1186/s12882-020-01951-1 32711468 PMC7382039

[18] Daniels N, Sabin JE Setting Limits Fairly: Learning to Share Resources for Health. 2nd ed Oxford University Press; 2008.

[19] Gunnar W The VA Transplant Program: a rebuttal to criticism and a look to the future. Am J Transplant. 2019;19(5):1288-1295. doi:10.1111/ajt.15295 30748088

[20] Gunnar W, Bronson DA, Cupples SA Access to transplant care and services within the Veterans Health Administration. Fed Pract. 2018;35(8):12-21. 30766376 PMC6263447

[21] Wong SPY, Vig EK, Taylor JS, Timing of initiation of maintenance dialysis: a qualitative analysis of the electronic medical records of a national cohort of patients from the Department of Veterans Affairs. JAMA Intern Med. 2016;176(2):228-235. doi:10.1001/jamainternmed.2015.7412 26809745 PMC4758379

[22] Wong SPY, McFarland LV, Liu C-F, Laundry RJ, Hebert PL, O’Hare AM Care practices for patients with advanced kidney disease who forgo maintenance dialysis. JAMA Intern Med. 2019;179(3):305-313. doi:10.1001/jamainternmed.2018.6197 30667475 PMC6439687

[23] Butler CR, Vig EK, O’Hare AM, Liu C-F, Hebert PL, Wong SPY Ethical concerns in the care of patients with advanced kidney disease: a national retrospective study, 2000-2011. J Gen Intern Med. 2020;35(4):1035-1043. doi:10.1007/s11606-019-05466-w 31654358 PMC7174459

[24] O’Hare AM, Butler CR, Taylor JS, Thematic analysis of hospice mentions in the health records of veterans with advanced kidney disease. J Am Soc Nephrol. Published online August 6, 2020:ASN.2020040473. doi:10.1681/ASN.2020040473 32764141 PMC7608965

[25] Braun V, Clarke V Using thematic analysis in psychology. Qual Res Psychol. 2006;3(2):77-101. doi:10.1191/1478088706qp063oa

[26] Birks M, Mills J. Grounded Theory: A Practical Guide. 2nd ed Sage Publications Inc; 2015.

[27] Giacomini MK, Cook DJ; Evidence-Based Medicine Working Group. Users’ guides to the medical literature: XXIII: qualitative research in health care A: are the results of the study valid? JAMA. 2000;284(3):357-362. doi:10.1001/jama.284.3.357 10891968

[28] Organ Procurement and Transplantation Network. Ethical principles in the allocation of human organs. US Department of Health and Human Services; 2015. Accessed May 23, 2018. https://optn.transplant.hrsa.gov/resources/ethics/ethical-principles-in-the-allocation-of-human-organs/

[29] Calestani M, Tonkin-Crine S, Pruthi R, ; ATTOM Investigators Patient attitudes towards kidney transplant listing: qualitative findings from the ATTOM study. Nephrol Dial Transplant. 2014;29(11):2144-2150. doi:10.1093/ndt/gfu188 24997006 PMC4209877

[30] Shpigel AA, Saeed MJ, Novak E, Alhamad T, Rich MW, Brown DL Center-related variation in cardiac stress testing in the 18 months prior to renal transplantation. JAMA Intern Med. 2019;179(8):1135-1136. doi:10.1001/jamainternmed.2019.0423 31081860 PMC6515568

[31] Akolekar D, Oniscu GC, Forsythe JLR Variations in the assessment practice for renal transplantation across the United Kingdom. Transplantation. 2008;85(3):407-410. doi:10.1097/TP.0b013e3181629bac 18322433

[32] Mandelbrot DA, Fleishman A, Rodrigue JR, Norman SP, Samaniego M Practices in the evaluation of potential kidney transplant recipients who are elderly: a survey of US transplant centers. Clin Transplant. 2017;31(10):e13088. doi:10.1111/ctr.13088 28805267

[33] Ladin K, Marotta SA, Butt Z, A mixed-methods approach to understanding variation in social support requirements and implications for access to transplantation in the United States. Prog Transplant. 2019;29(4):344-353. doi:10.1177/1526924819874387 31581889

[34] Hilbrands LB, Hoitsma AJ, Koene RA Medication compliance after renal transplantation. Transplantation. 1995;60(9):914-920. doi:10.1097/00007890-199511150-00006 7491693

[35] Sandhu GS, Khattak M, Woodward RS, Impact of substance abuse on access to renal transplantation. Transplantation. 2011;91(1):86-93. doi:10.1097/TP.0b013e3181fc8903 20966832

[36] Paterson TSE, O’Rourke N, Shapiro RJ, Loken Thornton W Medication adherence in renal transplant recipients: a latent variable model of psychosocial and neurocognitive predictors. PLoS One. 2018;13(9):e0204219. doi:10.1371/journal.pone.0204219 30265697 PMC6161882

[37] Ruzieh M, Foy AJ Time to rethink surveillance testing prior to kidney transplant: a teachable moment. JAMA Intern Med. 2020;180(10):1365-1366. doi:10.1001/jamainternmed.2020.2668 32776999

[38] Chapman EN, Kaatz A, Carnes M Physicians and implicit bias: how doctors may unwittingly perpetuate health care disparities. J Gen Intern Med. 2013;28(11):1504-1510. doi:10.1007/s11606-013-2441-1 23576243 PMC3797360

[39] Ku E, Lee BK, McCulloch CE, Racial and ethnic disparities in kidney transplant access within a theoretical context of medical eligibility. Transplantation. 2020;104(7):1437-1444. 31568216 10.1097/TP.0000000000002962 PMC7067676

[40] Berry KN, Daniels N, Ladin K Should lack of social support prevent access to organ transplantation? Am J Bioeth. 2019;19(11):13-24. doi:10.1080/15265161.2019.1665728 31647757

[41] Myaskovsky L, Almario Doebler D, Posluszny DM, Perceived discrimination predicts longer time to be accepted for kidney transplant. Transplantation. 2012;93(4):423-429. doi:10.1097/TP.0b013e318241d0cd 22228417 PMC3275654

[42] Ashford RD, Brown AM, Curtis B Substance use, recovery, and linguistics: the impact of word choice on explicit and implicit bias. Drug Alcohol Depend. 2018;189:131-138. doi:10.1016/j.drugalcdep.2018.05.005 29913324 PMC6330014

[43] Kasiske BL, Cangro CB, Hariharan S, ; American Society of Transplantation The evaluation of renal transplantation candidates: clinical practice guidelines. Am J Transplant. 2001;1(suppl 2):3-95. 12108435

[44] Center for Clinical Standards and Quality/Survey and Certification Group, Centers for Medicare and Medicaid Services Interpretive guidelines for the organ transplant conditions of participation (CoPs). US Department of Health and Human Services. Revised May 3, 2016. Accessed December 11, 2019. https://www.cms.gov/Medicare/Provider-Enrollment-and-Certification/SurveyCertificationGenInfo/Downloads/Survey-and-Cert-Letter-16-10.pdf

[45] Holm S What is wrong with compliance? J Med Ethics. 1993;19(2):108-110. doi:10.1136/jme.19.2.108 8331634 PMC1376198

[46] Sharif A The argument for abolishing cardiac screening of asymptomatic kidney transplant candidates. Am J Kidney Dis. 2020;75(6):946-954. doi:10.1053/j.ajkd.2019.05.033 31492488

[47] Gordon EJ, Butt Z, Jensen SE, Opportunities for shared decision making in kidney transplantation. Am J Transplant. 2013;13(5):1149-1158. doi:10.1111/ajt.12195 23489435

[48] US Government Accountability Office VA health care: additional training could improve organ transplant referral and evaluation processes. GAO publication 20-4. October 2019. Accessed December 11, 2019. https://www.gao.gov/assets/710/701897.pdf

