# Supplementary material for: Thematic Analysis of the Health Records of a National Sample of US Veterans With Advanced Kidney Disease Evaluated for Transplant
Source: JAMA Intern Med. 2020 Nov 23;181(2):212–9. doi: 10.1001/jamainternmed.2020.6388 (PMC7684522; doi:10.1001/jamainternmed.2020.6388)
Supplement: Supplement. — eFigure 1. Cohort Derivation eFigure 2. Diagram of Emergent Themes [file jamainternmed-e206388-s001.pdf]

Supplementary Online Content

Butler CR, Wightman A, Richards CA, et al. Thematic analysis of the health records of a national sample of US veterans with advanced kidney disease evaluated for transplant. *JAMA Intern Med*. Published online November 23, 2020. doi:10.1001/jamainternmed.2020.6388

eFigure 1. Cohort Derivation

eFigure 2. Diagram of Emergent Themes

This supplementary material has been provided by the authors to give readers additional information about their work.

© 2020 Butler CR et al. *JAMA Internal Medicine.*

eFigure 1. Cohort Derivation

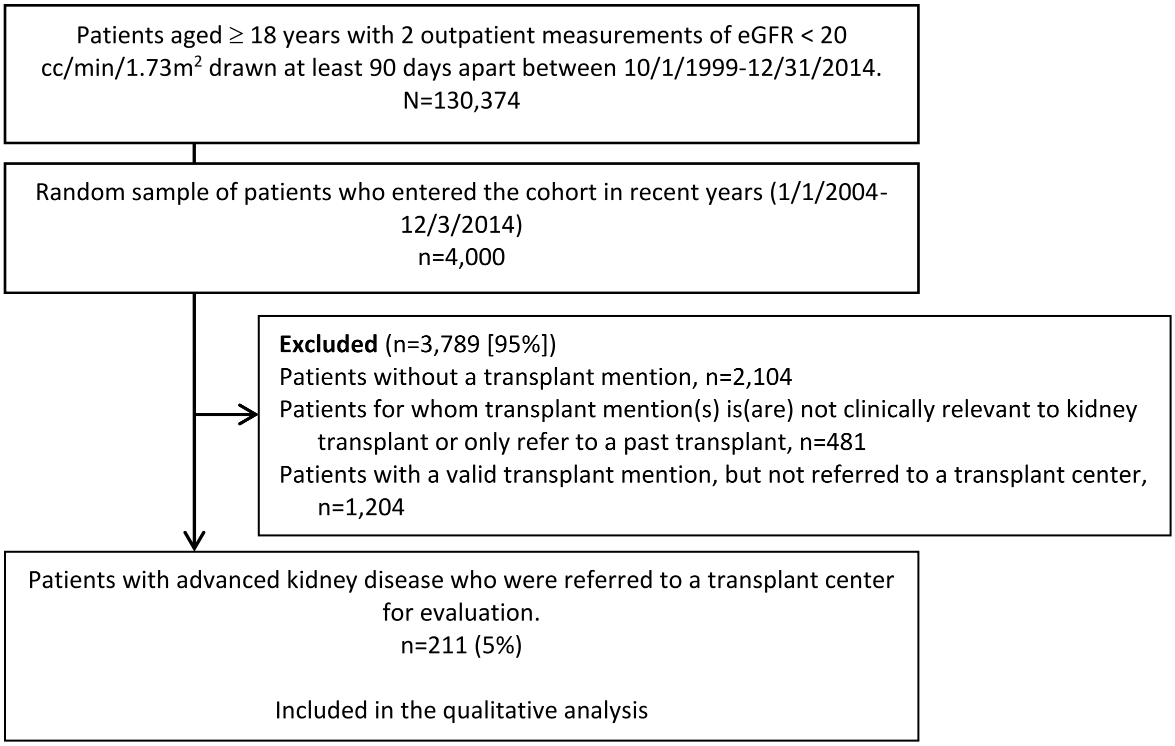

Abbreviation: eGFR, estimated glomerular filtration rate.

© 2020 Butler CR et al. *JAMA Internal Medicine.*

eFigure2. Diagram of Emergent Themes

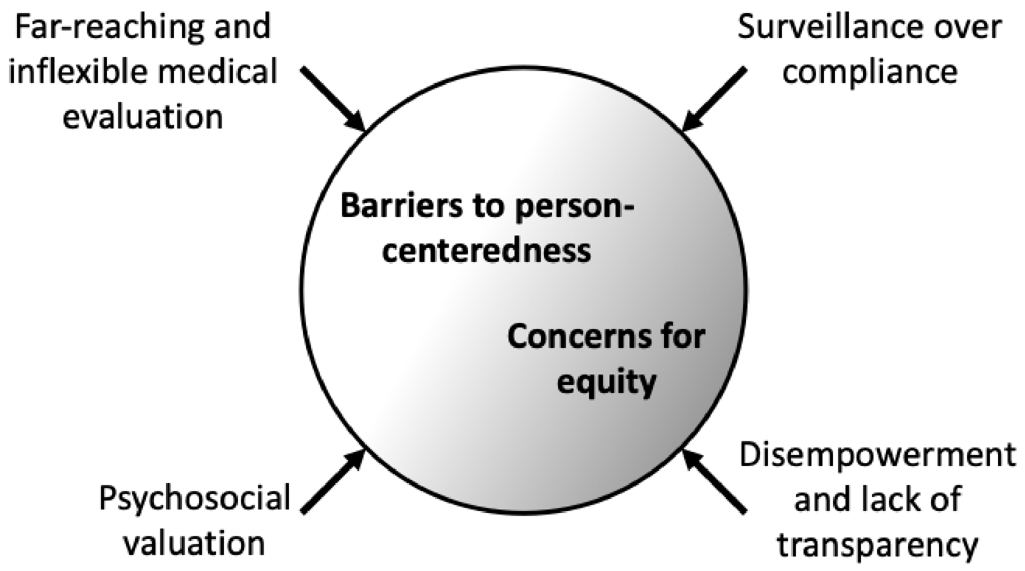

Four themes described practices and features of the kidney transplant evaluation that suggest that there are opportunities to make this process more person-centered and more equitable.

© 2020 Butler CR et al. *JAMA Internal Medicine.*
